# ACTH‐producing thymic neuroendocrine tumor initially presenting as psychosis: A case report and literature review

**DOI:** 10.1111/1759-7714.13099

**Published:** 2019-06-11

**Authors:** Taiki Okumura, Shohei Takayama, Shin‐ichi Nishio, Takahiro Miyakoshi, Takuro Noguchi, Takashi Kobayashi, Toshirou Fukushima, Nodoka Sekiguchi, Toshiaki Otsuki, Mitsuhisa Komatsu, Tomonobu Koizumi

**Affiliations:** ^1^ Second Department of Internal Medicine Shinshu University School of Medicine Asahi Matsumoto Japan; ^2^ Department of Comprehensive Cancer Therapy Shinshu University School of Medicine Asahi Matsumoto Japan; ^3^ Fourth Department of Internal Medicine Shinshu University School of Medicine Asahi Matsumoto Japan; ^4^ Department of Central Laboratory Shinshu University School of Medicine Asahi Matsumoto Japan

**Keywords:** Mediastinal carcinoid, mental disorder, neuroendocrine carcinoma, pulmonary metastasis, severe psychosis

## Abstract

A 32‐year‐old woman was referred to our hospital because of severe psychosis and was found to have an ectopic ACTH‐producing thymic neuroendocrine tumor. Laboratory data revealed an elevated serum cortisol and plasma ACTH level, hypokalemia, and metabolic alkalosis. Chest computed tomography (CT) revealed an anterior mediastinal mass and multiple pulmonary nodules. As the patient was unable to communicate because of her consciousness disturbance, she was managed with artificial ventilation and deep sedation. Metyrapone and potassium supplementation were administered, and steroid psychosis gradually improved. Thoracic surgery was performed and the histopathological diagnosis was thymic neuroendocrine tumor with positive anti‐ACTH immunohistochemical staining. Here we present details of the case and review the literature.

## Introduction

Ectopic adrenocorticotropic hormone (ACTH) secretion syndrome is a rare clinical manifestation, but is responsible for 15% of all cases of Cushing syndrome.^1^ The most common ectopic ACTH‐producing tumors are thoracic (bronchial and thymic) and gastroenteropancreatic neuroendocrine tumors (NET), followed by medullary thyroid carcinoma, small cell lung cancer, and pheochromocytoma.^2^, ^3^, ^4^

In general, the symptoms of Cushing syndrome include moon face, proximal muscle fatigue, skin pigmentation, uncontrolled hypertension, and diabetes. However, the intensity of symptoms in patients with ectopic ACTH‐producing tumors is dependent on the serum cortisol levels and the growth rate of the tumor. The clinical manifestations are varied in patients with ectopic ACTH‐producing tumors.^1^, ^2^, ^3^, ^4^

Psychiatric disturbances are frequently seen in patients with Cushing syndrome, the most common of which are depression and impaired cognition.^1^, ^5^ Severe psychosis related to increased cortisol secretion, such as emotional lability or mental disturbance, is rare, especially as the initial clinical manifestation of ectopic ACTH‐producing tumors.

We encountered a case of ectopic ACTH‐producing thymic NET initially presenting with severe psychosis that required artificial ventilation and deep sedation. Following improvement of psychosis with an inhibitor of adrenal corticosteroid synthesis, the patient continued treatment for the primary disease. Here, we describe the clinical course along with a brief review of the relevant literature.

## Case report

**Figure 1:**
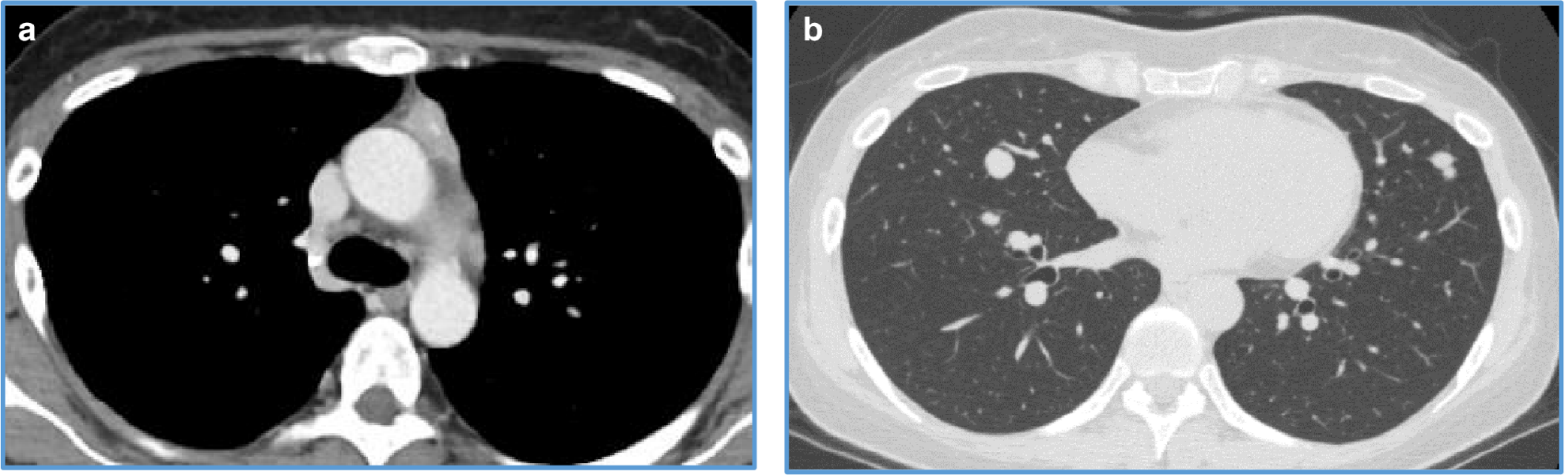
Computed tomography (CT) showed an anterior mediastinal mass (**a**), mediastinal lymph node and multiple pulmonary nodules (**b**).

**Figure 2:**
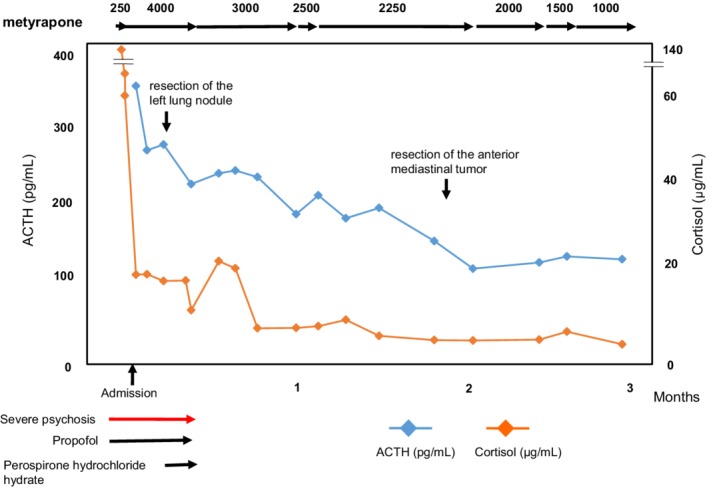
Alterations in serum cortisol and ACTH levels during the clinical course.

**Figure 3:**
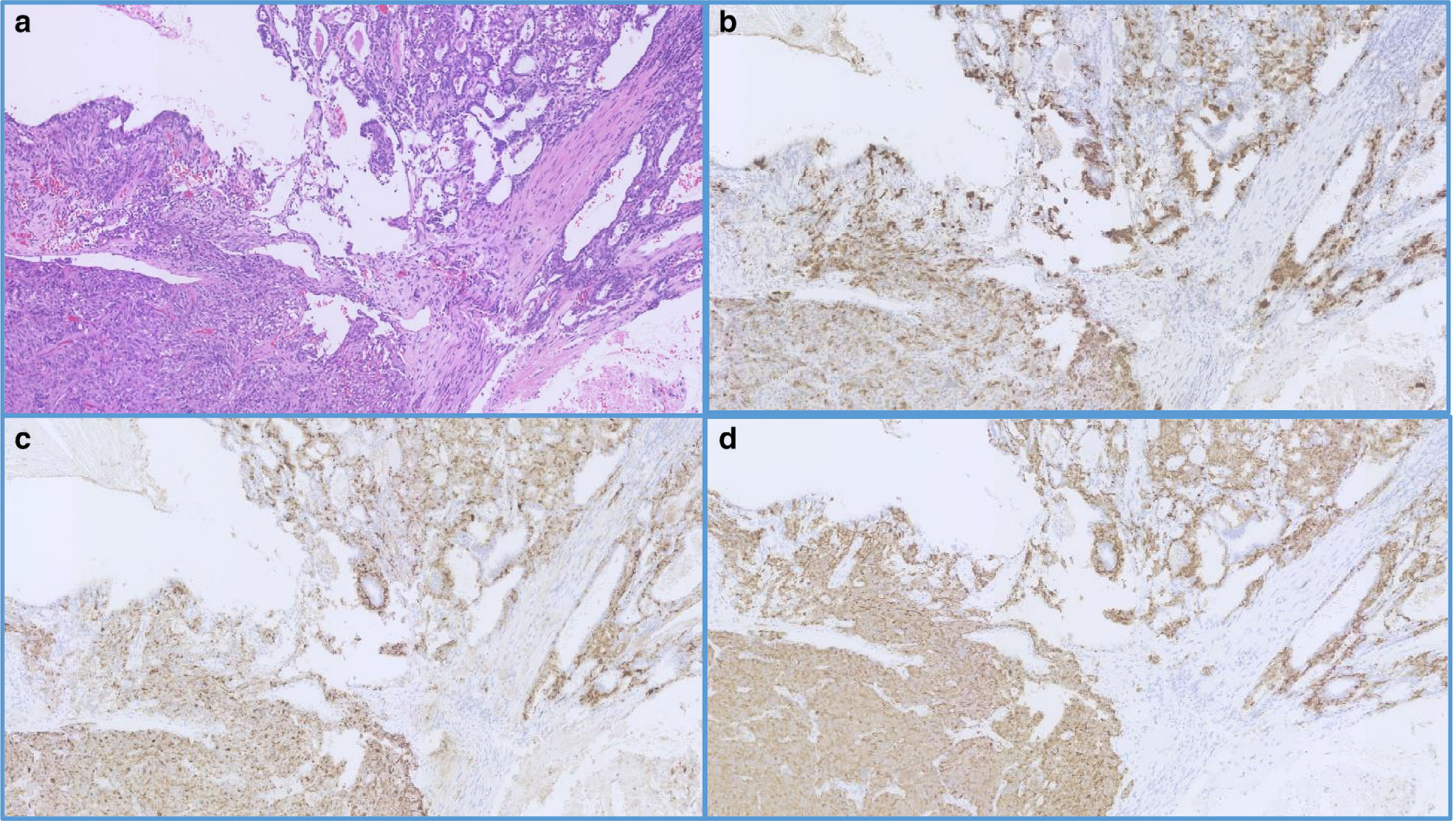
Hematoxylin and eosin staining of the resected specimen showed irregularly‐shaped sheets and nests of tumor cells (**a**). Immunohistochemical staining revealed tumor cells positive for ACTH (**b**), chromogranin A (**c**), and synaptophysin (**d**).

**Figure 4:**
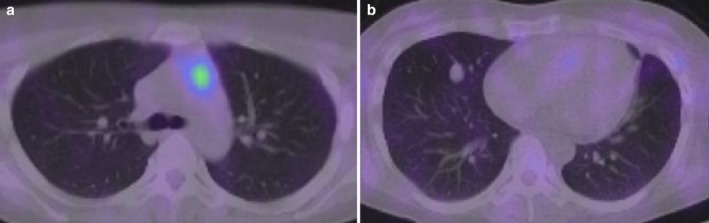
Somatostatin receptor scintigraphy showed abnormal accumulation in the anterior mediastinal mass (**a**) and pulmonary lesions (**b**).

A 32‐year‐old woman was referred to our hospital with severe psychosis. Three months previously, her family recognized emotional changes in the patient, such as euphoria and/or depression. She progressed to cognitive decline, insomnia, and whole‐body edema developed one week before visiting another hospital. On admission to the other hospital, initial endocrine investigations revealed high ACTH (545 pg/mL) and cortisol (136 μg/dL) levels. Computed tomography (CT) showed an anterior mediastinal mass and multiple pulmonary nodules (Fig 1a,b). As ectopic ACTH‐producing mediastinal tumor with pulmonary metastasis was suspected, metyrapone was administered at an initial dose at 250 mg/day, which was gradually increased to 750 mg/day. However, she lost the ability to communicate due to consciousness disturbance within one week of hospitalization. Therefore, she was transferred to the emergency unit of our hospital. Physical examination on admission revealed moon face and proximal muscle weakness. Body height and weight were 164 cm and 55 kg, respectively and her bodyweight had not altered during the previous few months. Blood pressure was 116/61 mmHg. She had emotional lability and impaired memory. Laboratory findings at our hospital demonstrated severe hypokalemia and metabolic alkalosis, in addition to the increase in serum ACTH and cortisol levels (Table 1). She showed self‐injurious behavior, and attempted to bite her tongue. Due to her severe psychosis and consciousness disturbance, intubation was required and she was sedated in the intensive care unit. Metyrapone was increased to the maximal dose of 4000 mg/day via nasogastric tube. In addition, potassium supplementation was performed at 200 mEq/day and spironolactone was used to control low potassium levels. On day 8, we added perospirone hydrochloride hydrate to propofol sedation to suppress restlessness (Fig 2). Thoracic surgery was performed for the lung nodule. Hematoxylin and eosin staining of the resected specimen indicated irregularly‐shaped sheets and nests of tumor cells, and a diagnosis of NET was made (Fig 3). Immunohistochemical staining revealed tumor cells positive for ACTH, chromogranin A, and synaptophysin. The Ki‐67 labeling index was <1%., indicating that cancer staging by the WHO classification was neuroendocrine tumor G1. The time course of changes in serum cortisol levels is shown in Figure 3. As cortisol levels decreased after initiation of metyrapone, her conscious disturbance improved. She became able to communicate and was extubated on day 14 of hospitalization. Somatostatin receptor scintigraphy was performed and showed abnormal accumulation in the anterior mediastinal mass (Fig 4a) but not in the pulmonary nodules (Fig 4b). There were no other metastatic organs, including the brain. Resection of the anterior mediastinal tumor and the largest metastatic lesion of the right lung were subsequently performed through a median sternotomy to decrease serum ACTH level as much as possible. The pathological findings, including immunological staining, in the mediastinal tumor were similar to the initially resected left lung tumor and confirmed that the tumor was thymic in origin. Thus, according to the WHO classification in 2018, a diagnosis of NET of the thymus (typical carcinoid) with multiple pulmonary metastases was made. Postoperatively, a combination of octreotide long‐acting repeatable (LAR; 30 mg) every four weeks and everolimus (10 mg/day) therapy has been continued, and ACTH and cortisol levels have remained stable. There was no personal or family history of multiple endocrine neoplasia type I in the present case.

**Table 1:** Laboratory data on admission to our hospital

Hematology			Hormone date		
WBC	8710	/*μ*L	ACTH	356.0	pg/mL
RRC	383	× 10^4^/*μ*L	Cortisol	65.3	μg/dL
Hb	14.1	g/dL	Free T3	1.54	pg/mL
PLT	14.2	× 10^4^/*μ*L	Free T4	1.30	ng/dL
Biochemistry			TSH	0.60	μIU/mL
TP	6.2	g/dL	ALD	53.4	pg/mL
Alb	3.7	g/dL	ARC	2.2	pg/mL
AST	28	IU/L	DHEA‐S	804.0	μg/dL
ALT	49	IU/L	*Blood gas*		
γGTP	35	U/L	pH	7.73	
T‐Bil	1.13	mg/dL	pO_2_	143	mmHg
ALP	141	U/L	pCO_2_	28.3	mmHg
LDH	390	U/L	HCO_3_^‐^	38.6	mEq/L
BUN	13.0	mg/dL	BE	16.7	mEq/L
Cre	0.49	mg/dL	Anion gap	9.5	mEq/L
Na	141	mEq/L			
K	2.8	mEq/L			
Cl	90	mEq/L			
CRP	0.07	mg/dL			

## Discussion

Here we report on a case of ectopic ACTH‐producing thymic NET. It is noteworthy that the patient developed hypercortisolemia‐induced psychosis that required artificial ventilation and deep sedation before diagnosis. The clinical manifestation of severe psychosis in ectopic ACTH‐producing tumors, especially at initial clinical presentation, is extremely rare in clinical practice.

Thymic NET accounts for approximately 2–4% of all anterior mediastinal tumors.^6^, ^7^ The prevalence of ectopic ACTH syndrome in all NETs was estimated to be 3.2%.^1^ The proportion in gastroenteropancreatic NETs was greater than thoracic (lung/bronchus and thymic) NETs. However, the prevalence of ectopic ACTH syndrome in thoracic NETs was significantly higher than that in gastroenteropancreatic NETs.^1^, ^3^ Thus, although ectopic ACTH‐producing thymic NET is extremely rare, the disease associated with ectopic ACTH syndrome should be noted in clinical practice. Based on the results of a PubMed search, Neary *et al.* 8 reported that the median age in patients with thymic NET associated with ACTH syndrome was 34 years, ranging from two to 75 years, and the male to female ratio was 1.5:1. Wick *et al.* 9 reported that thymic NET associated with ACTH syndrome had a higher 10‐year mortality rate than those without endocrinopathy (65% vs. 29%).^9^ Moran *et al.* 10 examined 80 NET patients and reported that patients with NET of the thymus and Cushing syndrome had a relatively poor prognosis.^10^ Based on a case series study, the median time to death was 35 months.^8^ In addition, several studies suggested that thymic NET associated with ACTH syndrome was an aggressive disease and/or had poor prognosis, particularly in young patients.^8^, ^10^, ^11^, ^12^

With regard to the psychiatric disturbance in patients with Cushing syndrome, insomnia, impaired cognition, memory disturbance, and depression have been reported at a frequency of <20%.^1^ Although our patient previously visited another hospital with insomnia and physical edema, she developed severe psychosis after hospitalization, resulting in the requirement for artificial ventilation and deep sedation. To our knowledge, only three similar cases have been reported previously.^13^, ^14^, ^15^ Similar to our case, psychotic symptoms were observed on diagnosis of ectopic ACTH syndrome in these previously reported cases. The serum cortisol level at diagnosis in the present case was higher than in the previous case reports. We have summarized case reports and reviewed patients with ACTH‐producing thymic tumors over the past 10 years published in English,^8^, ^15^, ^16^, ^17^, ^18^, ^19^, ^20^, ^21^, ^22^, ^23^, ^24^ and the cortisol and ACTH levels are presented in Table 2. Serum cortisol and plasma ACTH levels in our patient were relatively high, and we speculated that the extremely high cortisol level could have been related to the development of severe psychosis.

**Table 2:** Case reports and case series in ACTH‐producing thymic tumors over the past 10 years (published in English)

Case	Authors	Year of publication	Age	Sex	Tumor type	Cortisol (μg/dL)	ACTH (pg/mL)
1	Sato *et al.* 16	2010	56	F	Atypical thymic carcinoid	29	258
2	Saito *et al.* 17	2011	38	M	Large cell NEC of the thymus	34.1	140
3	Neary *et al.* 8	2012	Review (*n* = 12)		Thymic NET	—	149 (median)
4	Somasundarm *et al.* 18	2013	34	F	Thymic carcinoid	32.4	130
5	Barbieri *et al.* 19	2013	61	M	Typical thymic carcinoid	—	152
6	Sekiguchi *et al.* 20	2015	32	F	Thymic NET	39.2	68.7
7	Chen *et al.* 21	2016	Review (*n* = 16)		Atypical thymic carcinoid/typical thymic carcinoid	46.4 (median)	197 (median)
8	Oda *et al.* 22	2017	44	M	Large cell NEC of the thymus	49.1	354.1
9	Fujiwara *et al.* 23	2018	10	M	Typical thymic carcinoid	107.7	1100
10	Jibran *et al.* 12	2018	11	M	Thymic NET	28.2	105.1
11	Szczepanek‐Parulska *et al.* 24	2018	25	F	Thymic NET	63.4	268
12	Our case		32	F	Thymic NET	136	545

In addition, it is reported that the mean interval from the first clinical complaints to the diagnosis of ectopic ACTH syndrome was 11 months in patients with thymic NET,^1^ and the interval ranged from six months to eight years in other reports.^1^, ^3^, ^8^, ^11^ The interval in the present case was three months. The relatively short interval to onset and the markedly increased serum cortisol level may have been related to the development of psychotic symptoms in the present case. It is well known that the biological activities in thymic NETs are indolent, but hormonogenesis from the tumor cells may be intermittent or independent secretion with tumor growth.^8^, ^11^ Clinicians should be aware of the presence of psychosis disturbance at initial clinical presentation in patients with Cushing syndrome, although the physical signs, such as moon face, central obesity, and pigmentation of hypercortisolism are the predominant features.

Surgery is the most effective treatment for thymic NETs, even in patients with Cushing syndrome.^3^, ^6^, ^7^ However, it has been shown that thymic NETs exhibited more advanced or metastatic disease at the initial diagnosis compared with other NETs,^1^, ^3^, ^8^ which was also similar to our case. Therefore, medical management could play an important role in the treatment of thymic NETs. Based on large placebo‐controlled phase III studies, Frzio *et al.* 25 performed subgroup analysis and reported that everolimus improved progression‐free survival in cases of advanced lung/thymic NET by 2.4‐fold, compared with the placebo group. Our patient was treated with everolimus plus LAR and achieved stable disease. There is a lack of information or clinical experience available regarding the efficacy of chemotherapy in inoperable and metastatic thymic NET producing ACTH. Further case studies and/or clinical experience are required to determine the efficacy of chemotherapy.

In conclusion, we describe a case of ectopic ACTH‐producing thymic neuroendocrine tumor initially presenting with severe psychosis. ACTH‐producing thymic neuroendocrine tumor is extremely rare in clinical practice. However, we emphasize that steroid psychosis could be an initial presentation in patients with Cushing syndrome, and clinicians should be aware of possible mental disturbance as a clinical manifestation in patients with Cushing syndrome, as well as taking into account the results of general physical examinations.

## Disclosure

None of the authors have any potential conflicts of interest associated with this report.
